# Auditory and Visual Sustained Attention in Children with Speech Sound Disorder

**DOI:** 10.1371/journal.pone.0093091

**Published:** 2014-03-27

**Authors:** Cristina F. B. Murphy, Luciana O. Pagan-Neves, Haydée F. Wertzner, Eliane Schochat

**Affiliations:** Department of Physical Therapy, Speech-Language Pathology and Occupational Therapy, School of Medicine, University of São Paulo, São Paulo, Brazil; University of Leicester, United Kingdom

## Abstract

Although research has demonstrated that children with specific language impairment (SLI) and reading disorder (RD) exhibit sustained attention deficits, no study has investigated sustained attention in children with speech sound disorder (SSD). Given the overlap of symptoms, such as phonological memory deficits, between these different language disorders (i.e., SLI, SSD and RD) and the relationships between working memory, attention and language processing, it is worthwhile to investigate whether deficits in sustained attention also occur in children with SSD. A total of 55 children (18 diagnosed with SSD (8.11±1.231) and 37 typically developing children (8.76±1.461)) were invited to participate in this study. Auditory and visual sustained-attention tasks were applied. Children with SSD performed worse on these tasks; they committed a greater number of auditory false alarms and exhibited a significant decline in performance over the course of the auditory detection task. The extent to which performance is related to auditory perceptual difficulties and probable working memory deficits is discussed. Further studies are needed to better understand the specific nature of these deficits and their clinical implications.

## Introduction

Speech sound disorder is the most frequently occurring pediatric communication disorder [1]. The main characteristics of speech sound disorder involve difficulties in acquiring the phonological representation of speech sound systems and a limited capacity to accurately produce the sounds of the native speakers language [2], [3]. Recent studies have also demonstrated the presence of deficits in speech perception and in phonological tasks that assess phoneme awareness and phonological memory [4]–[6]. Poor phonological representations of speech sound systems are often attributed to deficits that involve phonological memory [5], [7], [8]. For example, Kenney et al [5] investigated the performance of adults with persistent developmental speech disorders on tests of verbal and non-verbal short term memory. The results of this study revealed that the speech disorders group performed significantly worse in nonverbal rhythm, tonal memory and verbal short-term memory.

It is also well-established that children with SSD are also at increased risks for specific language impairment [9], later reading disability (RD) and developmental dyslexia [10]–[12]. For example, in a longitudinal study, Peterson et al [12] investigated the relationship between childhood SSD and literacy development. These authors noted that children with histories of SSD are more likely to develop reading disorders. Broomfield and Dodd [9] also found strong bidirectional comorbidity between SSD and LI based on referrals to pediatric speech and language therapy services. Recent genetic studies have also shown that RD, SSD and ADHD share the same genetic locus at 6p22 [13]–[15]. This relationship could lead to related phenotypic characteristics and an overlap of cognitive symptoms among these three disorders [16].

Given this expected cognitive overlap and the high comorbidity of attention deficit hyperactivity disorder (ADHD) and language disorders [17], recent research has investigated whether attention deficits are also overlapping symptoms of SLI and RD [18]–[23]. Finneran et al [18] investigated the performance of 4- to 6-year-old children with SLI on a visual sustained attention task. When compared to typically developing children (TD), the children with SLI were significantly less accurate but not significantly slower than the TD children on the test of visual sustained attention. In a study conducted by Spaulding et al [19], children with SLI also exhibited poorer performance than did typically developing children on an auditory sustained attention task, which supports the idea that children with SLI exhibit attention difficulties. Spaulding et al [19] found no differences between the groups in visual sustained attention tasks. Ebert and Kohnert [20] performed a meta-analysis and concluded that deficits in sustained attention seem to be part of the language impairment profile, particularly in tasks that use auditory-linguistic stimuli. Several studies have also confirmed the existence of attention deficits in children with dyslexia [21]–[23]. For example, Facoetti and Molteni [22] showed that children with dyslexia have difficulties in focusing attention on text and simultaneously inhibiting distractor stimuli.

No study has investigated sustained attention in children with SSD; however, given the presence of sustained attention deficits in children with SLI and RD and the well-established relationship between SLI, SSD and RD, it is worthwhile to examine whether this deficit also occurs in children with SSD. According to Gomes et al [24], all components of attention have a role in language acquisition; for example, to accurately identify and interpret incoming linguistic information, sustained attention is required to maintain focus on the speech input, to attend to relevant information and to ignore irrelevant information [18]. Moreover, because attention is closely related to phonological working memory, it is plausible that attention plays a role in language processing.

The current research aims to examine the performance of children with SSD on auditory and visual sustained attention tests. As in most of the studies of children with SLI and RD [18], [20], this study assessed attention using continuous performance test protocols. These protocols assess performance with tasks that require the participant to remain prepared to respond to an infrequent target (e.g., digits, letters or symbols) over an extended period of time and measure both the maintenance of attention and inhibitory control. The outcome measures are the number of targets detected (HITS), the number of false alarms meaning incorrect responses to nontarget stimuli (FAs) and the response times (RTs). In terms of sensory modalities, Spaulding et al [19] found that the attention deficits of children with SLI are restricted to auditory stimuli. This finding was confirmed by Ebert and Kohnert [20] in a meta-analysis. In contrast, Finneran et al [18] found deficits for visual stimuli. Due to this controversy, we assessed both sensory modalities in the present study to provide more details about the performance of children with SSD in attention tests.

## Methods

### Ethics statement

This study was conducted at the Department of Physical Therapy, Speech-Language Pathology and Occupational Therapy in the School of Medicine at the University of Sao Paulo and was approved by the Research Ethics Committee of the Analysis of Research Projects of the Hospital das Clínicas, Medicine School, University of São Paulo, under protocol number 575/09. A written consent form with detailed information about the aim and the protocols of the study was also approved by this ethics committee. All parents provided written informed consent on behalf of their children prior to participation in the study.

### Participants

A total of 55 children were invited to participate in this study: 18 diagnosed with SSD (SSD group) and 37 typically developing children (TD group). The children in the SSD group were recruited through the Laboratory of Investigation in Phonology within the departments of Physical Therapy, Speech-Language Pathology and Audiology and Occupational Therapy of the School of Medicine at the University of São Paulo and diagnosed using the Phonology [25] and Vocabulary Test [26] from the Infantile Language Test-ABFW [27]. The phonology test consists in a picture naming task (with 34 words corresponding to 90 consonants) and a task with imitation of single words (with 39 words corresponding to 107 consonants). The Percentage of Consonants Correct- Revised (PCC-R) [28] index was calculated separately for both tasks [29]–[31]. None of these children were undergoing rehabilitation. The children in the TD group were recruited from the general community. The inclusion criteria for this group were the absence of speech sound errors on the Phonology Test from the ABFW, normal hearing and no previous speech-language interventions. The children in both groups were monolingual Brazilian-Portuguese learners.

The inclusion criteria common to both groups were as follows: aged between 7 and 12 years; IQ > 80 (based on the WISC-IV); and no familial or personal history of diagnosed or suspected auditory, otological or neurological disorder or injuries. Additionally, the participants were required to demonstrate normal tympanometric and acoustic reflexes and symmetrical auditory sensitivity within normal limits (≤ 15 dB HL for octave frequencies from 250 to 8000 Hz and ≤ 5 dB HL at any interaural frequency difference). After recruitment, both groups were required to pass a screening battery that consisted of the following: a medical history interview, an audiological evaluation, non-verbal auditory perceptual measures (i.e., the Frequency Pattern Test [32] and Gap in Noise Test [33]), language tests (the ABFW Test – Picture Naming and Repetition of Words), a short-term memory test (the Digit Span Test) and a non-verbal IQ test (the RAVEN Test of Coloured Progressive Matrices with Brazilian norms [34] and a conversion table for IQ values [35]). The language test (i.e., the ABFW Test) was also administered to the TD group to confirm the absence of speech sound disorders.

### Procedures and measures

After the groups were established, a series of tests was applied to investigate the attention skills of both groups.

In the present research, two attention tests were developed using E-Prime Professional Software. The protocols of these tests were loosely modeled on previously published tests [36], [37], and the length of the task and the protocol parameters were also based on previously published protocols [38]–[40].

**Figure 1:**
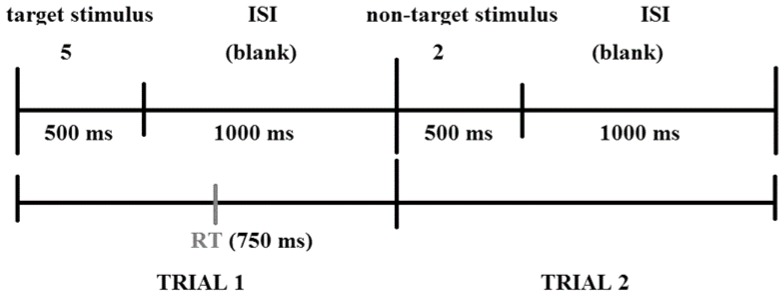
Example of two trials of the visual test: the first with a target stimulus (5) and the response time (RT) of 750 ms; the second with a non-target stimulus (2). ISI: inter-stimulus interval.

In the visual test, digits between 1 and 7 were presented on a screen, and the participants were instructed to press a button as quickly as possible every time a 1 or a 5 appeared. The auditory task was identical to the visual version except that the participants heard the digit spoken over a set of calibrated headphones. The auditory stimuli consisted of recordings of digits between 1 and 7 being read in a quiet recording studio by a female adult whose native language was Brazilian Portuguese (phonetic transcription of the stimuli: ['uw, 'do^j^s, 'tejs, 'k^w^at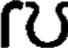, 'sijk, 'se^j^s, 'sεt∫i]). The duration of the recorded digits was fixed at approximately 500 msec regardless of the number of syllables in the numeral. The stimuli were presented binaurally at a comfortable listening level that corresponded to a sound pressure level of 70 dB (A). The duration of each test was approximately 6 minutes, and each test consisted of 210 trials that were divided into 3 blocks of 70. The division of the tests into 3 blocks enabled us to investigate the vigilance decline effect, which measures the decline in performance over the course of the experiment. For each trial, the digit appeared during the first 500 msec of the trial and was followed by an inter-stimulus interval of 1000 msec (Figure 1). Three performance measures were compared across blocks: HITs, FAs and RTs (i.e., response times).

Participants were tested individually in a quiet, well-lit laboratory on campus. The order of the visual and auditory tests was counterbalanced across participants. Before each test, each participant was given the appropriate instructions and asked to perform approximately 15 practice trials.

### Statistical analyses

The data were analyzed using SPSS version 18.0 (SPSS, Chicago, IL). Repeated-measures ANOVAs were performed to analyze intra- and inter-group (TD and SSD) differences. Mauchly’s sphericity test was used to test the assumption that the data were spherical, and post hoc analyses (Bonferroni) were performed to identify the mean performance measures that differed significantly. The Kolmogorov-Smirnov test was used to assess the normality of the data from each group, and the data were normalized using a logarithmic transformation. Pearson’s correlations were calculated to determine the relationship between performance on the attention and memory tests. According to Dancey & Reidy [41], r = 0.10 to 0.30 demonstrates a weak correlation, r = 0.40 to 0.60 demonstrates a moderate correlation, and r = 0.70 to 1.0 demonstrates a strong correlation.

## Results

### Screening battery performance

The results of the initial test battery and the inclusion criteria (i.e., the IQ test and the audiological evaluation) led to the exclusion of 2 children from the SSD group and 4 children from TDT group. Table 1 displays the characteristics of both groups including mean age, gender, language, IQ, short-term memory and audiological performance. Comparisons between groups were performed using analyses of variance (ANOVAs).

**Table 1:** Performance characteristics of the SSD and TD groups on the screening battery.

Variables	SSD (n = 18)	TD (n = 37)	p
Gender (n)			
Girls (%)	33,3	44	
Boys (%)	66,6	56	
Age (M ± DP)	8.11±1.2	8.76±1.4	0.012
Language tasks (M ± DP)			
Picture naming (%)	80.34±12.17	100±0.0	**<0.001**
Repetition of words (%)	80.27±12.33	100±0.0	**<0.001**
Short-term memory			
Digit Span	3.83±0.61	5.43±1.14	**<0.001**
Auditory tests			
Audiological evaluation	No alteration	No alteration	
Frequency Pattern Test	41.1±16.93	73.1±22.29	**<0.001**
Gap in Noise Test	7.13±2.35	4.5±0.7	**<0.001**
IQ score (Raven test)	106.47±8.1	111.41±16.4	0.265

Analyses of variance (ANOVAs) revealed significant between-group differences in the language tests (repetition [F(3,35) = 18.795, p<0.001, n2 = 0.617] and naming [F(3,35) =  20.764, p<0.001, n2 = 0.640]), in the Digit Span test [F (1,53)  = 40.02, p<0.001, η2 = 0.43] and in both of the auditory perceptual tasks [Frequency Pattern (p<0.001) and GIN (p<0.001)]. No differences were found in age [F(1,53) =  2.50, p<0,12] or IQ (p = 0.115).

### Attention tests

Table 2 displays the performance of both groups on the visual and auditory attention tests in terms of HITs, FAs and RTs.

**Table 2:** Means and Standard Deviations of the Performance Measures for All Experimental Conditions for both groups.

*Measures*	*Group*	*Mean*	*SD*	*F*	*p-value*	*η^2^*
Visual HIT	TD	55,92	7,7	0,2	0,62	
	SSD	56,44	2,5			
Visual False Alarme	TD	2,38	3,2	2,3	0,13	
	SSD	4,17	5,458			
Visual RT	TD	645,72	98,2	3,0	0,09#	
	SSD	692,67	85,7			
Auditory HIT	TD	46,03	10,3	0,2	0,62	
	SSD	44,67	7,5			
Auditory False Alarm	TD	5,30	4,0	5,93	0,02*	0,10
	SSD	9,00	7,1			
Auditory RT	TD	1104,93	73,9	3,33	0,07#	
	SSD	1065,13	79,7			

Analyses of variance (ANOVAs) revealed a significant between-group difference in one of the auditory attention test measures; i.e., the auditory false alarm measure [F(1,53)  = 5.93, p = 0.02, η2 = 0.10].

**Figure 2:**
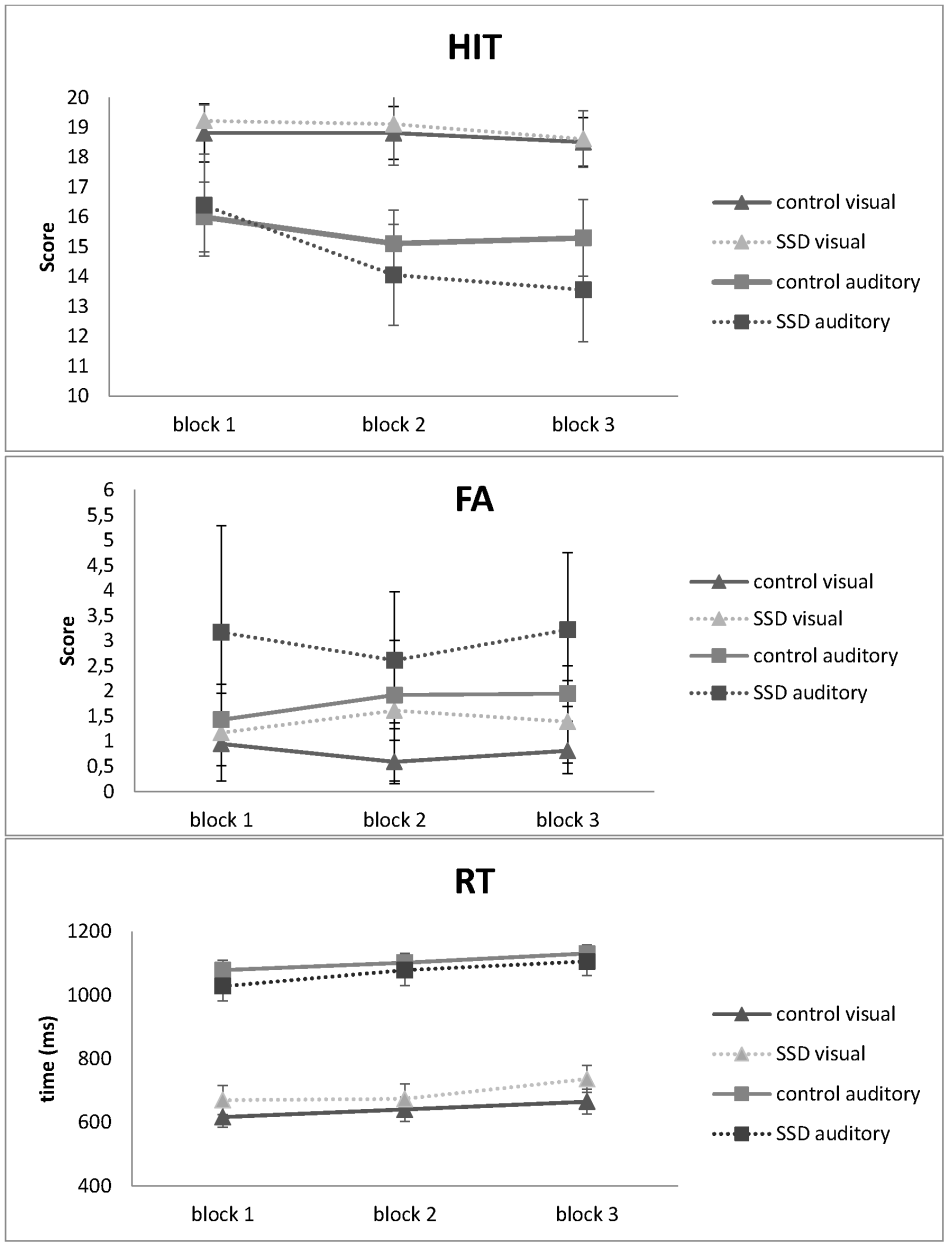
Performance along blocks for both groups. FA (false alarm); RT (response time).

Figure 2 displays the results from all auditory and visual attention tests for both groups. Differences across the three blocks in all measures (i.e., HIT, FA and RT) were analyzed.

There were no significant declines in performance in terms of HIT rate in either the visual [F(2,72)  = 1.03, p = 0.36] or auditory modalities [F(2,72) =  2.66, p = 0.08] in the TD group. In the SSD group, there was no significant decline in the visual modality [F(2,34) =  0.44, p = 0.64]; however, in contrast to the results of the TD group, the SSD group exhibited a significant decline in HIT rate in the auditory modality [F(2,34) =  6.55, p<0.01]. Post-hoc analyses using Bonferroni’s adjustment for multiple comparisons revealed that the differences between blocks 1 and 2 (p = 0.02) and between blocks 1 and 3 (p<0.01) were significant.

The false alarm rates were similar for both modalities and both groups; there were no significant differences in performance across blocks in either group (TD group–visual [F(2,72) =  1.37, p = 0.26] and auditory [F(2,72) =  2.63, p = 0.08]; SSD group–visual [F(2,34) =  0.40, p = 0.67] and auditory [F(1,45, 24.83)  = 0.23, p = 0.72]. However, although neither group exhibited declines in performance across blocks, the SSD group performed significantly worse than did the TD group in terms of auditory false alarms (FA block 1 [F(1,53)  = 5.93, p = 4.85, η2 = 0.08] and FA block 3 [F (1,53)  = 4.04, p = 0.05, η2 = 0.07]).

Response time increased significantly for both groups in both modalities: TD group–visual [F (2.72)  = 7.98, p<0.01, η2 = 0.18] and auditory [F (2,72)  = 8.93, p<0.01, η2 = 0.2]; SSD group–visual [F (2,34)  = 15.47, p<0.01, η2 = 0.48] and auditory [F (2,34) = 5.52, p<0.01, η2 = 0.25.] Post-hoc analyses using Bonferroni’s adjustment for multiple comparisons revealed that, in the TD group, blocks 1 and 3 were different in the visual modality (p<0.001) and that there was a trend toward significance between blocks 1 and 2 in the auditory modality (p = 0.06). In the SSD group, blocks 1 and 3 in the visual modality were different (p<0.01), and blocks 2 and 3 in the auditory modality were different (p<0.01). Although both groups exhibited significant declines in performance in this specific measure, there was no group effect, which indicates that the changes in performance across the blocks of the tests were similar across groups (visual RT [F(1,53)  = 3.57, p>0.05, r = 0.06] and auditory RT [F(1,53) = 2.33, p>0.05, r = 0.04]). Indeed, the only significant difference in RT occurred in the last block of the auditory modality [F (1,53)  = 5.13, p = 0.03, η2 = 0.09].

**Figure 3:**
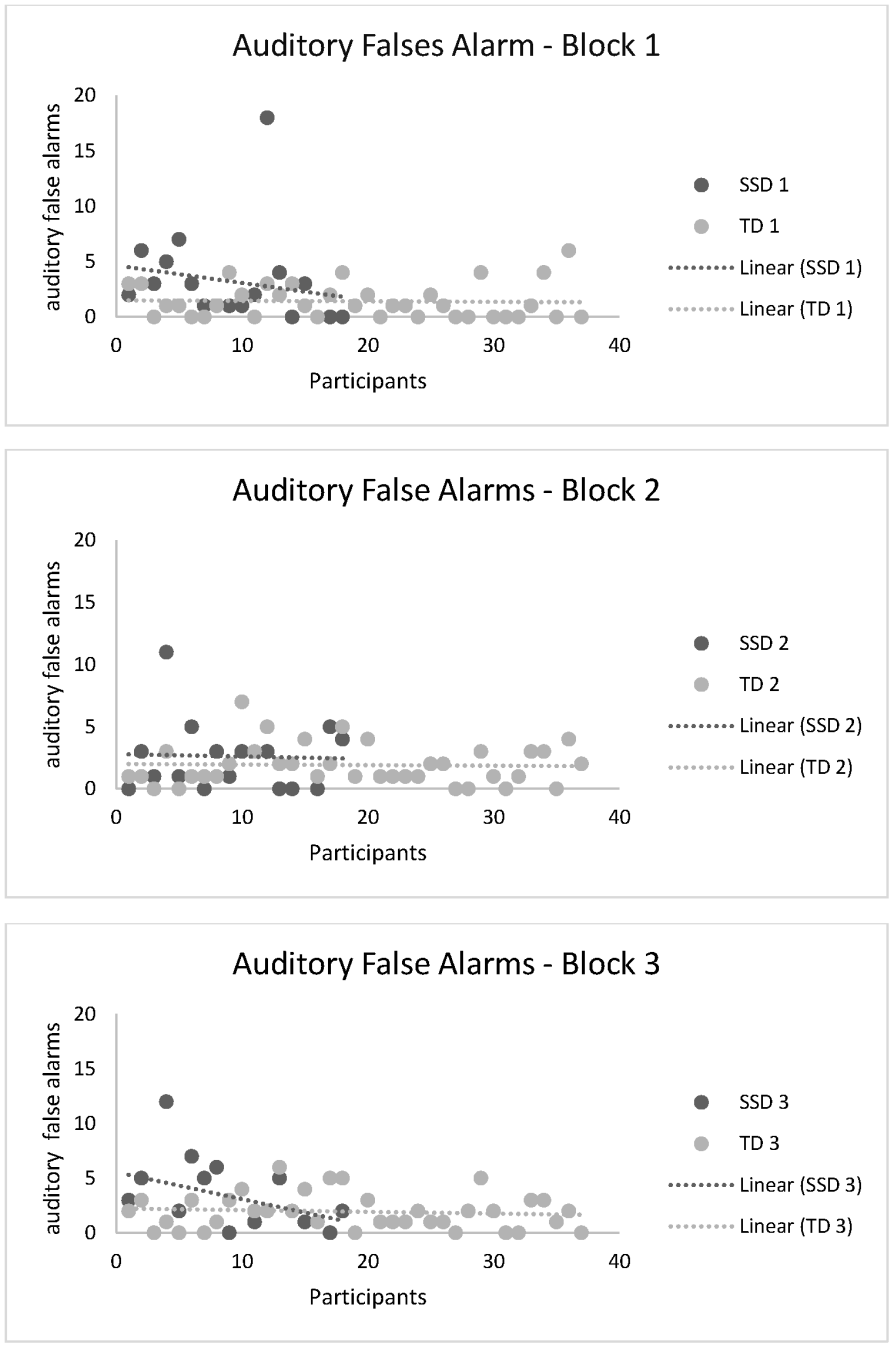
Distribution of the participants by the number of auditory false alarms.

Given the worse performance of the SSD group in terms of auditory false alarms, we also included the Figure 3, with the distribution of the participants by the number of auditory false alarms. In all blocks, the comparison between both trend lines demonstrate the superior performance of the most participants of the TD group, compared with the SSD group.

### Correlations

Tables 3 show the correlations between the auditory, language, memory and attention performance measurements.

**Table 3:** Correlation between performance measures of TD and SSD group.

	Age		Digit Span		FPT		GIN		Naming		Repetition		Visual HIT		Visual FA		Visual RT		Auditory HIT		Auditory FA	
	TD	SSD	TD	SSD	TD	SSD	TD	SSD	TD	SSD	TD	SSD	TD	SSD	TD	SSD	TD	SSD	TD	SSD	TD	SSD
Digit Span	0.19	0.10							_		_											
FPT	0.49*	–0.11	0.43*	–0.40					_		_											
GIN	–0.38*	–0.49*	0.036	–0.14	–0.42*	0.03			_		_											
Naming	___	0.04	___	0.07	__	0.28	___	0.07	_		_											
Repetition	___	0.12	___	0.13	__	0.24	___	0.13	_	0.96*	_											
Visual HIT	0.06	0.27	0.16	–0.18	0.08	–0.10	0.16	–0.13	_	–0.00	_	–0.00										
Visual FA	–0.15	0.03	–0.21	–0.06	–0.21	0.03	0.16	0.28	_	–0.02	_	0.06	–0.23	–0.12								
Visual RT	–0.27	–0.43	–0.30	–0.05	–0.49*	–0.12	0.46*	0.46*	_	–0.05	_	–0.03	0.08	–0.08	0.40*	–0.18						
Auditory HIT	0.23	0.47*	0.32*	0.18	0.46*	0.07	–0.55*	–0.18	_	0.33	_	0.34	0.59*	0.43*	–0.03	–0.16	–0.39*	–0.42				
Auditory FA	–0.43*	–0.01	–0.28	0.23	–0.30	–0.29	0.37*	0.02	_	–0.27	_	–0.27	0.05	–0.24	0.54*	0.50*	0.41*	–0.20	–0.25	–0.26		
Auditory RT	–0.06	–0.24	–0.14	–0.01	–0.22	0.15	0.18	0.34	_	0.12	_	0.17	0.26	0.27	0.14	–0.53*	0.72*	0.16	–0.21	0.34	0.08	–0.69

For the TD group, there was a moderate, negative correlation between auditory FA and age, visual RT and FPT, Auditory HIT and GIN. There was also a moderate, positive correlation between Auditory HIT and FPT, visual RT and GIN. For the SSD group, there was a moderate, positive correlation between visual RT and GIN.

## Discussion

The purpose of the present study was to assess auditory and visual sustained attention in children with SSD. First, it is important to discuss the performance characteristics of the SSD and TD groups on the screening battery. As expected, the SSD group exhibited poorer performances on both language tests (i.e., the picture naming and repetition of words tests). Moreover, the SSD group also exhibited poorer performances on the short-term memory test and both of the auditory perceptual tests (i.e., the Frequency Pattern Test and the GIN Test). In terms of memory abilities, our results corroborate those of a previous study in that both studies found that SSD children exhibited poorer short-term memory performance as measured with the digit span task [13]. The authors of this previous study suggested that children with SSD and children with language or reading impairments have difficulties in developing memory traces of speech input that are sustained for a sufficient duration to abstract acoustic features for phonological encoding. Regarding auditory perceptual performance, no study has applied the tests used in the present research to investigate the auditory perceptual skills of children with SSD; however, several researchers have found the same nonverbal auditory perception difficulties in children with specific language delays and dyslexia [42], [43]. In summary, the performance of both groups in the language and memory tests confirms the significantly worse language skills of the SSD group compared to the TD group.

Based on the 3 measures analyzed, the SSD exhibited poorer sustained attention for auditory but not visual stimuli as indicated by a significant decline in HITs as the test progressed. The SSD group also demonstrated a greater number of false alarms in both the visual and auditory modality when compared to the TD comparison group.

As both between-group differences occurred in the auditory modality, we hypothesize that these results are related to the poorer performances of the SSD group in both auditory perceptual measures. Thus, the nonverbal auditory perceptual impairments of the children with SSD might lead to difficulties in properly discriminating auditory stimuli. Consequently, these children may adopt a strategy of anticipating the stimuli, which would produce numerous false alarms. Moreover, the significant decline in auditory HIT performance over the course of the test suggests that the task was more difficult for these children in the auditory modality and thus caused fatigue. These results corroborate the findings of Spaulding et al [19] that demonstrated that children with SLI perform poorly on auditory sustained attention tasks compared to typically developing children. Furthermore, as in the present study, Spaulding et al found no differences in visual sustained attention tasks. Finneran et al [18] also noted that, because children with SLI have impairments in auditory processing skills (which may negatively affect task performance), auditory attention tests should not be used. Therefore, similar to the studies that have been conducted on SLI groups [18], [19], in the present research, the poorer performance of the SSD group might reflect auditory processing impairments and not sustained attention impairments per se. Despite that, no significant correlation was found between auditory false alarms and both auditory tasks in the current research. Given that the present auditory tasks (FP and GIN) are specifically related to the auditory temporal processing, further studies should investigate others aspects of auditory processing.

Another hypothesis regarding the poorer performance of the SSD group is related to impulsivity skills. According to Riccio et al [44], false responses are an indication of impulsivity; therefore, the current results might suggest that the impulse control skills of the SSD group were less mature. No study has investigated this specific behavior in children with SSD, but it is well-documented that younger children exhibit more impulsive behavior than do older children. Furthermore, Diamond [45] argued that the poor performance of young children could be attributed to a combination of insufficient working memory and immature inhibitory functioning skills that are associated with the prefrontal region. In a recent review, Bari and Robbins [46] also noted that several fMRI and PET studies have associated deficits in go/no-go tasks with the medial portion of the prefrontal cortex; this region is also associated with working memory skills [47]. While attempting to construct a model to understand the behavior of ADHD children, Barkley [48] noted that poor behavioral inhibition (like that observed in ADHD) might lead to secondary deficits in working memory and associated functions. Working memory load also seems to be associated with declines in performance, such as the decline in auditory HIT performance observed in the present research. According to Davies and Parasuraman [49], such decrements in vigilance represent deteriorations in accuracy or detection speed over time. These declines occur primarily when stimulus events are presented rapidly in successive discrimination tasks as in the current research. According to Parasuraman [50], in such situations, decrements in sensitivity over time are linked to working memory requirements and the depletion of attentional resources throughout the period of vigilance. The current study did not investigate working memory performance in children with SSD; therefore, further studies are needed to better understand whether these possible immature behaviors and decrements in vigilance decrement are associated with working memory impairments in children with SSD.

The current study had some limitations. The sample size of the study group was relatively small, which may reduce the generalizability of the results of this study. Furthermore, both attention tasks measured sustained attention abilities. Therefore, further studies should consider larger samples and investigate other aspects of attention, such as selective attention.

In conclusion, the children with SSD performed worse on the auditory attention test; these children produced more false alarms and exhibited a significant decline in HIT performance over the course of the auditory test. The extent to which performance is related to difficulties in auditory perceptual abilities and probable working memory deficits is unclear. Further studies are necessary to better understand the nature of this poor performance and its clinical consequences in terms of rehabilitation.

## Conclusion

Children with SSD disorders exhibited poorer performance on the auditory attention test than did the TD group; the children with SSD produced a greater number of false alarms, and their auditory HIT performance declined significantly over the course of the test. Further studies are needed to better understand the specific nature of these deficits and their clinical implications.
